# Increased bleeding events with the addition of apixaban to the dual anti-platelet regimen for the treatment of patients with acute coronary syndrome: A meta-analysis

**DOI:** 10.1097/MD.0000000000025185

**Published:** 2021-03-26

**Authors:** Jing Jin, Xiaojun Zhuo, Mou Xiao, Zhiming Jiang, Linlin Chen, Yashvina Devi Shamloll

**Affiliations:** a Department of Cardiology, The Fourth Hospital of Changsha, Changsha, Hunan; b Department of Cardiology, Hospital of Northwestern Polytechnical University, Xi an, Shanxi; c Tongji Medical College, Huazhong University of Science and Technology, Wuhan, Hubei, PR China.

**Keywords:** acute coronary syndrome, anticoagulant, apixaban, bleeding events, dual antiplatelet therapy

## Abstract

**Background:**

Dual anti-platelet therapy (DAPT) with aspirin and clopidogrel has been the mainstay of treatment for patients with acute coronary syndrome (ACS). However, the recurrence of thrombotic events, potential aspirin and clopidogrel hypo-responsiveness, and other limitations of DAPT have led to the development of newer oral anti-thrombotic drugs. Apixaban, a new non-vitamin K antagonist, has been approved for use. In this meta-analysis, we aimed to compare the bleeding outcomes observed with the addition of apixaban to DAPT for the treatment of patients with ACS.

**Methods:**

Online databases including EMBASE, Cochrane Central, http://www.ClinicalTrials.gov, MEDLINE and Web of Science were searched for English based publications comparing the use of apixaban added to DAPT for the treatment of patients with ACS. Different categories of bleeding events and cardiovascular outcomes were assessed. The analysis was carried out by the RevMan software version 5.4. Odds ratios (OR) with 95% confidence intervals (CI) were used to represent the data following analysis.

**Results:**

This research analysis consisted of 4 trials with a total number of 9010 participants. Thrombolysis in myocardial infarction (TIMI) defined major bleeding (OR: 2.45, 95% CI: 1.45–4.12; *P* = .0008), TIMI defined minor bleeding (OR: 3.12, 95% CI: 1.71–5.70; *P* = .0002), International society of thrombosis and hemostasis (ISTH) major bleeding (OR: 2.49, 95% CI: 1.80–3.45; *P* = .00001) and Global Use of Strategies to Open Occluded Arteries (GUSTO) defined severe bleeding (OR: 3.00, 95% CI: 1.56–5.78; *P* = .01) were significantly increased with the addition of apixaban to DAPT versus DAPT alone in these patients with ACS. However fatal bleeding (OR: 10.96, 95% CI: 0.61–198.3; *P* = .11) was not significantly different.

**Conclusions:**

Addition of the novel oral anticoagulant apixaban to the DAPT regimen significantly increased bleeding and therefore did not show any beneficial effect in these patients with ACS. However, due to the extremely limited data, we apparently have to rely on future larger studies to confirm this hypothesis.

## 1 Introduction

Dual anti-platelet therapy (DAPT) with aspirin and clopidogrel has been the mainstay of treatment for patients with acute coronary syndrome (ACS).^[1–2]^ However, the recurrence of thrombotic events despite DAPT use,^[3]^ the development of aspirin or clopidogrel hypo-responsiveness^[4]^ and other limitations of DAPT have led to the development of newer oral anti-thrombotic drugs.^[5]^

Even if the antiplatelet agents have significant antithrombotic effects, major limitations have been observed with current antiplatelet drugs.^[6]^ Aspirin resistance refers to the inability of aspirin to fully inhibit platelet activities.^[7]^ Studies have shown that in spite of their antiplatelet treatment, 10% to 20% of patients with a history of an ischemic event develop recurrent events following an acute myocardial infarction (MI) or stroke. Recent studies have also shown clopidogrel hyporresponsiveness^[8]^ which might have been due to concomitant clinical conditions such as diabetes mellitus,^[9]^ platelet hyperactivities,^[10]^ low fibrinolytic potential, an increased platelet turn-over, and the administration of certain drugs which might interact and decrease the effect of antiplatelets. In addition, a variety of polymorphisms in the CYP2C19 gene has shown to also contribute to clopidogrel hyperresponsiveness.^[11]^ Nevertheless, newer antiplatelet agents have been able to address some but not all the limitations.

Previous studies have shown beneficial effects of triple anti-platelet therapy (TAPT) to an extent when compared to DAPT in patients with ACS.^[12–14]^ Triple antiplatelet therapy was associated with significantly reduced restenosis and target vessel revascularization. However, the safety side of TAPT with cilostazol^[15]^ or warfarin^[16]^ as the third antithrombotic drug was questionable. While warfarin in TAPT was apparently associated with a significantly higher bleeding risk, cilostazol was associated with higher adverse events leading to drug discontinuation.

Recently, several novel oral anti-thrombotic drugs have been approved for use by the Food and Drug Administration (FDA).^[17]^ Dabigatran and rivaroxaban have already been used in patients with heart diseases.^[18]^ Apixaban, another non-vitamin K antagonist, is a new oral antithrombotic drug^[19]^ which might be added to DAPT to form a new TAPT regimen.

In this meta-analysis, we aimed to compare the bleeding outcomes observed with the addition of apixaban to DAPT for the treatment of patients with ACS.

## 2 Methods

### 2.1 Search databases and search strategies

The most accessible online databases including EMBASE, Cochrane Central, http://www.ClinicalTrials.gov, MEDLINE and Web of Science were searched for English based publications (until November 2020) comparing the use of apixaban added to DAPT for the treatment of patients with ACS.

During this search process, the following terms or phrases were used:

- apixaban and acute coronary syndrome;
- apixaban and myocardial infarction;
- apixaban and coronary artery disease;
- apixaban and dual anti-platelet therapy;
- apixaban and percutaneous coronary intervention;
- apixaban and aspirin and clopidogrel.

The abbreviations ACS, CAD (coronary artery disease), PCI (percutaneous coronary intervention), DAPT were also interchanged during the search process.

The inclusion criteria were studies that:

1. compared the use of apixaban in addition to DAPT for the treatment of patients with ACS;
2. reported bleeding events and/or adverse cardiovascular outcomes;
3. consisted of relevant data which could be used in this meta-analysis.

The exclusion criteria included:

1. systematic reviews and meta-analyses; literature reviews, letter to editors, and case studies;
2. studies that did not involve the addition of apixaban to DAPT;
3. studies that did not report the relevant endpoints;
4. studies that involved data which were irrelevant to this meta-analysis;
5. studies that repeated themselves in other databases (duplicated studies).

### 2.2 Data extraction

Six authors were involved in the data extraction process. After having carefully assessed the data from relevant trials, the total number of participants assigned to the apixaban and control groups respectively, the anti-platelet agents which were used, the total number of events which were associated with each subgroup of outcomes, the age, gender, the co-morbidities present, the types of participants, were extracted and formulated in tables.

Any disagreement which occurred during the data extraction process was resolved by a careful discussion with the corresponding author.

### 2.3 Methodological quality appraisal

The methodological qualities of the trials were assessed by the authors based on the recommendations suggested by the Cochrane Collaboration tool.^[20]^ Grades were allotted; grade A being associated with a low risk of bias, grade B with a moderate risk, and grade C with a high risk of bias. Each author allotted a fair score and an average score was then calculated and recorded.

### 2.4 Outcomes reported in the studies

Majority of the participants had ST elevation myocardial infarction (STEMI) and non-ST elevation myocardial infarction (NSTEMI).

The follow-up time periods which were reported in the original studies varied from 6 months to 1.8 years as shown in Table 1.

**Table 1:** Outcomes reported in the original studies.

Study	Types of participants	Outcomes reported	Follow up time period
APPRAISE 2^[25]^	ACS including STEMI and NSTEMI and UA	MACEs, all-cause mortality, cardiac death, MI, stroke, stent thrombosis, TIMI major and minor bleeding, ISTH major and minor bleeding, GUSTO major and minor bleeding, fatal bleeding, intracranial bleeding, any bleeding	8 months
APPRAISE J^[25]^	ACS including STEMI and NSTEMI	ISTH major and minor bleeding, any bleeding	6 months
APPRAISE^[26]^	ACS including STEMI and NSTEMI	ISTH major bleeding, any bleeding, TIMI defined major and minor bleeding	6 months
ARISTOLE^[27]^	PCI + AF	Stroke, MI, all-cause death, ISTH major bleeding	1.8 years

ACS = acute coronary syndrome, AF = atrial fibrillation, GUSTO = global use of strategies to open occluded arteries, ISTH = International society on thrombosis and hemostasis, MACEs = major adverse cardiac events, MI = myocardial infarction, NSTEMI = non-ST elevation myocardial infarction, PCI = percutaneous coronary intervention, STEMI = ST elevation myocardial infarction, TIMI = thrombolysis in myocardial infarction.

Table 1 also lists the outcomes which were reported in each of the original studies.

The following endpoints were assessed in this analysis:

1. Endpoints which were related to bleeding events included;
2. Thrombolysis in myocardial infarction (TIMI) defined major and minor bleedings;^[21]^
3. International society of thrombosis and hemostasis (ISTH) defined major and minor bleedings;^[22]^
4. Global Use of Strategies to Open Occluded Arteries (GUSTO) defined bleeding;^[23]^
5. Any bleeding event;
6. Fatal bleeding.

Endpoints related to the adverse cardiovascular outcomes included:

1. Major adverse cardiac events (MACEs);
2. All-cause mortality;
3. Myocardial infarction (MI);
4. Stroke;
5. Stent thrombosis.

### 2.5 Statistical analysis

This is a meta-analysis of randomized controlled trials. The most appropriate software to analyze the data was the RevMan software version 5.4. Odds ratios (OR) with 95% confidence intervals (CI) were used to represent the data following analysis.

Heterogeneity assessment was carried out by the Q statistic test. A subgroup analysis with a *P* value less or equal to .05 was considered statistically significant whereas a *P* value greater than .05 was considered statistically insignificant in this study.

Heterogeneity assessment was also dependent on the value of *I*^*2*^ which was generated during the analysis. Heterogeneity was increased with an increasing *I*^*2*^ value, whereas a low *I*^*2*^ value denoted a low heterogeneity.

For an analysis with a low heterogeneity, a fixed statistical effect model was used, whereas a random statistical effect model was used for an analysis with a high heterogeneity.

Sensitivity analysis was also carried out. Publication bias was observed by visually assessing the funnel plots.

### 2.6 Compliance with ethical guidelines

This is a meta-analysis involving data which were extracted from previously published original studies. Therefore, ethical approval or board review approval was not required.

## 3 Results

### 3.1 Search outcomes

Following a careful search (PRISMA reporting guideline),^[24]^ a total number of 212 publications were obtained. The authors carefully assessed the titles and abstracts and nonrelevant studies were immediately eliminated (176 studies). Thirty six full-text articles were assessed for eligibility.

The full text articles were thoroughly assessed, and further eliminations were carried out based on the following:

- Literature reviews based on novel oral anti-thrombotic agents (2);
- Case studies (3);
- Rationale of future trial (1);
- Did not involve relevant data which could be used in this analysis (2);
- Duplicated studies or studies which involved the same trials (24).

**Figure 1:**
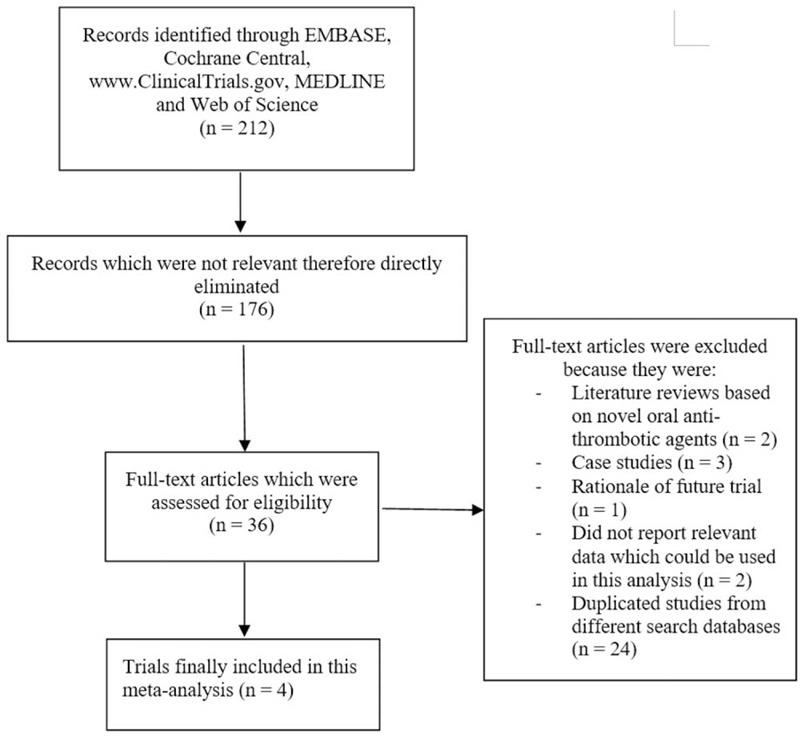
Flow diagram representing the study selection based on the PRISMA guideline.

Finally, only 4 trials^[25–27]^ were confirmed for this meta-analysis as shown in Figure 1.

### 3.2 Trial characteristics

This research analysis consisted of 4 trials with a total number of 9010 participants (enrolled between the years 2006–2010). 4508 participants were treated with DAPT plus apixaban whereas 4502 participants were treated with DAPT alone (placebo group). Details involving the total number of participants which were extracted from each study have been listed in Table 2.

**Table 2:** Main characteristics of the studies.

Study	Patients’ enrollment time period	Total No of participants assigned to apixaban (n)	Total No of participants assigned to placebo (n)	Type of study	Bias risk grade
APPRAISE 2^[25]^	2009–2010	3705	3687	RCT	B
APPRAISE J^[25]^	2009	99	52	RCT	B
APPRAISE^[26]^	2006–2007	630	599	RCT	B
ARISTOLE^[27]^	2006–2010	152	164	RCT	B
Total No of participants (n)		4508	4502		

RCT = randomized controlled trials.

After an assessment of the methodological quality of each original trial, a grade ‘B’ was finally allotted.

### 3.3 Baseline features of the participants

Table 3 lists the baseline characteristics of the participants. Majority of the participants were male patients with a mean age varying from 60.0 to 71.0 years. Study ARISTOLE consisted of the eldest participants in comparison to the other studies with a mean age of 71 years, followed by study APPRAISE 2 whereby the participants had a mean age of 67 years. Study APRAISE consisted of the youngest participants with a mean age ranging from 60 to 61.5 years. Study APPRAISE J consisted of 89.9% of male participants in the experimental group and 80.8% of participants in the control group whereas study APPRAISE 2 consisted of the lowest number of male participants with a mean percentage of 67.4% in the experimental group and 68.3% in the control group. The percentage of participants with co-existing diabetes mellitus was ≤50%. Study APPRAISE consisted of the lowest number of participants with diabetes mellitus. Percentage of patients with heart failure also varied from 7.70% to 40.2% and those patients with prior stroke varied from 0.00% to 10.2% as shown in Table 3.

**Table 3:** Baseline features of the participants in each group.

Study	Age	Males	T2DM	HBP	CVE	HF
	Exp/Cntl	Exp/Cntl	Exp/Cntl	Exp/Cntl	Exp/Cntl	Exp/Cntl
APPRAISE 2	67.0/67.0	67.4/68.3	48.7/47.0	65.7/65.3	10.2/9.90	40.2/40.1
APPRAISE J	65.0/63.9	89.9/80.8	34.4/50.0	–	6.10/0.00	10.05/7.70
APPRAISE	61.5/60.0	72.7/74.3	22.1/23.2	–	4.60/4.90	16.9/9.70
ARISTOLE	71.0/71.0	77.0/76.2	–	85.5/92.7	7.20/8.50	24.3/27.4

Cntl= control group (non-apixaban), CVE = cerebrovascular events, Exp = experimental group (apixaban), HBP = high blood pressure, HF = heart failure, T2DM = type 2 diabetes mellitus. Age was reported in years, whereas the other features were reported in percentage (%).

Table 4 lists the antithrombotic medications with specific dosages which were used by the participants in the experimental as well as the placebo groups.

**Table 4:** The anti-thrombotic medications.

Study	Experimental group	Placebo group
APPRAISE 2	Apixaban 5 mg twice daily + ASA + clopidogrel	ASA + clopidogrel
APPRAISE J	Apixaban 2.5 mg or 5 mg twice daily + ASA + clopidogrel	ASA + clopidogrel
APPRAISE	Apixaban 2.5 mg twice daily or 10 mg 6 hourly + ASA + clopidogrel	ASA + clopidogrel
ARISTOLE	Apixaban 2.5 mg or 5 mg twice daily + ASA + clopidogrel	Warfarin + or ASA + or clopidogrel

ASA = aspirin.

### 3.4 Main result comparing the addition of apixaban to DAPT versus placebo

**Figure 2:**
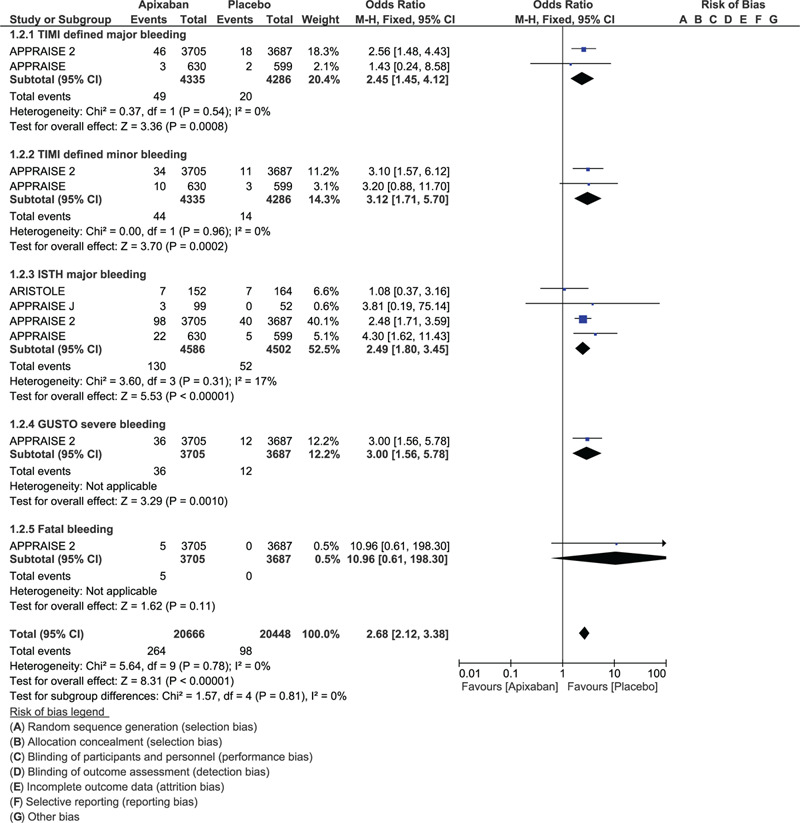
Bleeding events observed with the addition of apixaban to DAPT vs DAPT alone in patients with acute coronary syndrome (Part I).

**Figure 3:**
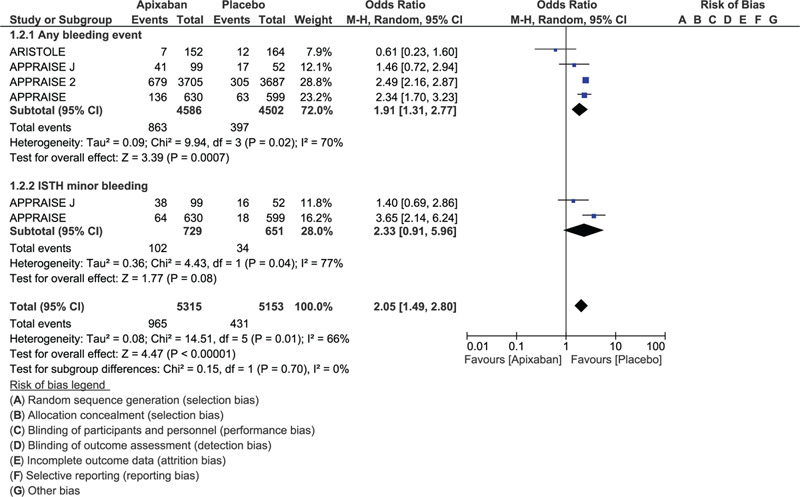
Bleeding events observed with the addition of apixaban to DAPT vs DAPT alone in patients with acute coronary syndrome (Part II).

This analysis showed TIMI defined major bleeding (OR: 2.45, 95% CI: 1.45–4.12; *P* = .0008), TIMI defined minor bleeding (OR: 3.12, 95% CI: 1.71–5.70; *P* = .0002), ISTH major bleeding (OR: 2.49, 95% CI: 1.80–3.45; *P* = .00001) and GUSTO defined severe bleeding (OR: 3.00, 95% CI: 1.56–5.78; *P* = .01) to be significantly increased with the addition of apixaban to DAPT versus DAPT regimen alone in these patients with ACS as shown in Figure 2. Any bleeding event (OR: 1.91, 95% CI: 1.31–2.77; *P* = .0007) was also significantly increased with the addition of apixaban (Fig. 3). However fatal bleeding (OR: 10.96, 95% CI: 0.61–198.3; *P* = .11) and ISTH defined minor bleeding (OR: 2.33, 95% CI: 0.91–5.96; *P* = .08) were not significantly different (Figs. 2 and 3).

**Figure 4:**
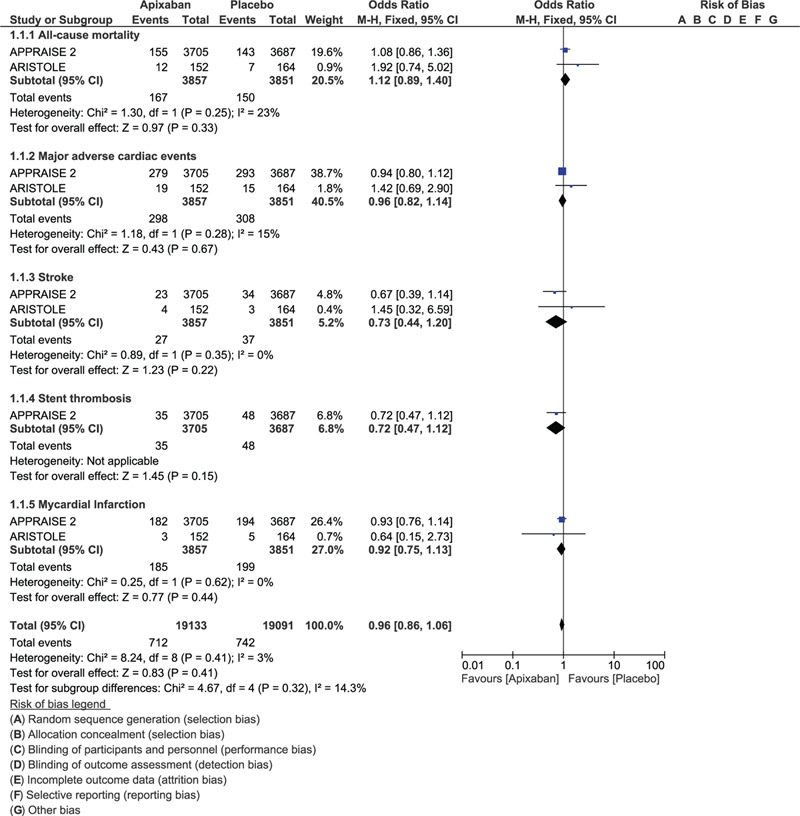
Adverse cardiovascular outcomes with the addition of apixaban to DAPT vs DAPT alone in patients with acute coronary syndrome.

When the cardiovascular outcomes were assessed, no significant change was observed in all-cause mortality (OR: 1.12, 95% CI: 0.89–1.40; *P* = .33), MACEs (OR: 0.96, 95% CI: 0.82–1.14; *P* = .67), stroke (OR: 0.73, 95% CI: 0.44–1.20; *P* = .22), stent thrombosis (OR: 0.72, 95% CI: 0.47–1.12; *P* = .15), and MI (OR: 0.92, 95% CI: 0.75–1.13; *P* = .44) when apixaban was added to DAPT as shown in Figure 4.

The results have been summarized in Table 5.

**Table 5:** Results of this analysis.

Endpoints	OR with 95% CI	*P* value
TIMI defined major bleeding	2.45 [1.45–4.12]	.0008
TIMI defined minor bleeding	3.12 [1.71–5.70]	.0002
ISTH major bleeding	2.49 [1.80–3.45]	.00001
ISTH defined minor bleeding	2.33 [0.91–5.96]	.08
GUSTO defined severe bleeding	3.00 [1.56–5.78]	.01
Any bleeding event	1.91 [1.31–2.77]	.0007
Fatal bleeding	10.96 [0.61–198.3]	.11
All-cause mortality	1.12 [0.89–1.40]	.33
MACEs	0.96 [0.82–1.14]	.67
Stroke	0.73 [0.44–1.20]	.22
Stent thrombosis	0.72 [0.47–1.12]	.15
MI	0.92 [0.75–1.13]	.44

CI = confidence intervals, GUSTO = global use of strategies to open occluded arteries, ISTH = International society on thrombosis and hemostasis, MACEs = major adverse cardiac events, MI = myocardial infarction, OR = odds ratios, TIMI = thrombolysis in myocardial infarction.

**Figure 5:**
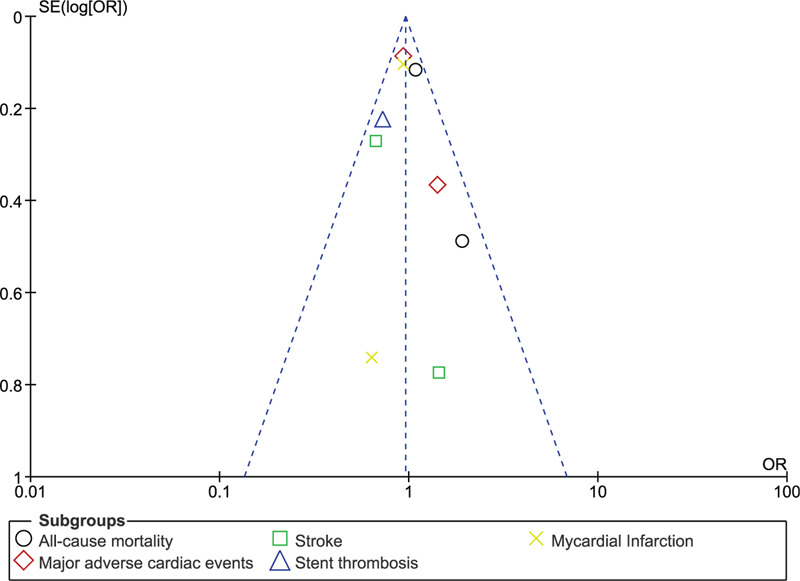
Funnel plot representing publication bias (A).

**Figure 6:**
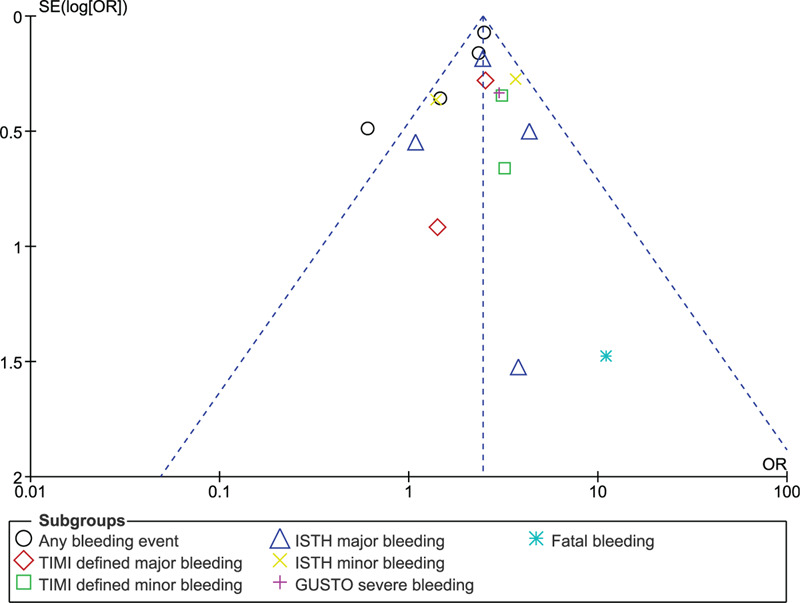
Funnel plot representing publication bias (B).

Consistent results were obtained throughout based on the sensitivity analysis carried out. In addition, Figures 5 and 6 showed a low evidence of publication bias.

## 4 Discussion

Addition of the novel oral anti-coagulant apixaban to DAPT in patients with ACS was tested in this analysis. A brief mechanism of action of this novel antithrombotic agent has been reported.^[28]^

Briefly, this drug, with a mechanism of action which is different from aspirin^[29]^ and clopidogrel,^[30]^ works by inhibiting factor Xa of the coagulation cascade, and thus indirectly decreases the formation of clot induced by thrombin. It was approved for use by the FDA in December 28, 2012, for the prevention of stoke in patients with atrial fibrillation. Then 2 years later, it was approved for the treatment of pulmonary embolism and deep vein thrombosis.

Results of this analysis showed the addition of apixaban to DAPT not to have any beneficial effect. In fact, the addition of apixaban was associated with significant bleeding events when compared to the use of DAPT alone. TIMI defined minor and major bleeding events, GUSTO defined severe bleeding, ISTH major bleeding was all significantly increased, without any significant change in adverse cardiovascular outcomes.

Majority of the participants were extracted from the Apixaban for Prevention of Acute Ischemic Events 2 (APPRAISE-2) trial. Similar to this current pooled analysis, results from the APPRAISE-2 trial also showed the addition of apixaban to DAPT to be associated with significantly higher bleeding events.^[31]^ Its concomitant use with aspirin alone was also not beneficial. Apixaban significantly increased TIMI defined major bleeding in patients taking aspirin [1.48 versus 0.25, adjusted hazard ratio (HR): 6.62, 95% CI: 0.75 to 51.73] and in patients who were taking DAPT (aspirin and clopidogrel) [2.58 vs 1.02; adjusted HR: 2.44; 95% CI: 1.34 – 4.45, *P* = .41]. Another study based on the APPRAISE-2 trial showed apixaban to be associated with an increased bleeding tendency in patients with or without heart failure.^[32]^ Patients with acute heart failure had a significantly increased rate of TIMI defined major bleeding with apixaban. However, numerically fewer clinical events were observed with apixaban compared to placebo, a trend which was not reported in patients with prior heart failure or no heart failure.

Even if the use of oral anticoagulants and their associated outcomes in patients with ACS were poorly described, a recent study from the American Heart Association showed that patients treated with chronic oral anticoagulants experienced greater in-hospital bleeding which required readmission.^[33]^ It should be noted that the study included data from an integrated health care system from years 2009 to 2014. Of the 9566 PCIs which were carried out, 8.8% of the participants were on oral anticoagulants, of which, 7.9% were using nonvitamin K antagonists. After revascularization, patients who were treated with oral anticoagulants had higher crude rates of major bleeding, access and nonaccess site bleedings. This was also shown in a meta-analysis comparing triple therapy versus DAPT.^[16]^ However, the only difference was that vitamin K antagonist was used in contrast to our current analysis which included non-vitamin K antagonist as the anticoagulant.

Nevertheless, the inclusion of newer oral anticoagulants with DAPT is quite challenging and selection of antithrombotic agents should be made at individual level in patients with ACS.^[34]^ Until now, DAPT still remains the antithrombotic regimen of choice for ACS patients.

### 4.1 Limitations

Similar to several other studies, a lack of participants represented the first limitation of this analysis. Secondly, not all the endpoints were reported in the original studies. If trial A reported endpoints s, t, w, x, y, and z; trial B reported only x, y, and z; and trial C reported only s, w, and z. Therefore, we could not include all the studies each time when assessing the endpoints and this could be another limitation of this analysis. Another limitation could be the dosage of apixaban which was used. One study reported the use of 10 mg 6 hourly whereas the other studies reported a dosage of 2.5 or 5 mg 12 hourly. This might have influenced the outcomes. Also, 1 study consisted of participants with the use of warfarin in the placebo group. In addition, even if most of the studies consisted of participants with ACS, there was 1 study which included patients with atrial fibrillation undergoing percutaneous coronary intervention. Moreover, there was a variation in the follow-up time period reported in each original trial. Another limitation was the fact that there was less information available on the duration of antithrombotic treatment in these patients.

## 5 Conclusions

Addition of the novel oral anticoagulant apixaban to the DAPT regimen significantly increased bleeding and therefore did not show any beneficial effect in these patients with ACS. However, due to the extremely limited data, we apparently have to rely on future larger studies to confirm this hypothesis.

## Author contributions

**Conceptualization:** Jing Jin, Xiaojun Zhuo, Mou Xiao, Zhiming Jiang, Linlin Chen, Yashvina Shamloll.

**Data curation:** Jing Jin, Xiaojun Zhuo, Mou Xiao, Zhiming Jiang, Linlin Chen, Yashvina Shamloll.

**Formal analysis:** Jing Jin, Xiaojun Zhuo, Mou Xiao, Zhiming Jiang, Linlin Chen, Yashvina Shamloll.

**Funding acquisition:** Jing Jin, Xiaojun Zhuo, Mou Xiao, Zhiming Jiang, Linlin Chen, Yashvina Shamloll.

**Investigation:** Jing Jin, Xiaojun Zhuo, Mou Xiao, Zhiming Jiang, Linlin Chen, Yashvina Shamloll.

**Methodology:** Jing Jin, Xiaojun Zhuo, Mou Xiao, Zhiming Jiang, Linlin Chen, Yashvina Shamloll.

**Project administration:** Jing Jin, Xiaojun Zhuo, Mou Xiao, Zhiming Jiang, Linlin Chen, Yashvina Shamloll.

**Resources:** Jing Jin, Xiaojun Zhuo, Mou Xiao, Zhiming Jiang, Linlin Chen, Yashvina Shamloll.

**Software:** Jing Jin, Xiaojun Zhuo, Mou Xiao, Zhiming Jiang, Linlin Chen, Yashvina Shamloll.

**Supervision:** Jing Jin, Xiaojun Zhuo, Mou Xiao, Zhiming Jiang, Linlin Chen, Yashvina Shamloll.

**Validation:** Jing Jin, Xiaojun Zhuo, Mou Xiao, Zhiming Jiang, Linlin Chen, Yashvina Shamloll.

**Visualization:** Jing Jin, Xiaojun Zhuo, Mou Xiao, Zhiming Jiang, Linlin Chen, Yashvina Shamloll.

**Writing – original draft:** Jing Jin, Xiaojun Zhuo, Mou Xiao, Zhiming Jiang, Linlin Chen, Yashvina Shamloll.

**Writing – review & editing:** Jing Jin, Xiaojun Zhuo, Mou Xiao, Zhiming Jiang, Linlin Chen, Yashvina Shamloll.
